# Genome-Wide Analysis of *AAT* Genes and Their Expression Profiling during Fiber Development in Cotton

**DOI:** 10.3390/plants10112461

**Published:** 2021-11-15

**Authors:** Dongjie Yang, Yuanyuan Liu, Hailiang Cheng, Qiaolian Wang, Limin Lv, Youping Zhang, Dongyun Zuo, Guoli Song

**Affiliations:** 1 Institute of Cotton Research of Chinese Academy of Agricultural Sciences, Anyang 455000, China; 82101185040@caas.cn (D.Y.); yuanyl4956@163.com (Y.L.); pser2010@163.com (H.C.); wangql1232021@163.com (Q.W.); llm0372@126.com (L.L.); zyp547550790@163.com (Y.Z.); 2 Zhengzhou Research Base, State Key Laboratory of Cotton Biology, Zhengzhou University, Zhengzhou 450001, China

**Keywords:** *AAT*, gene family, fiber, gene expression profile, cotton

## Abstract

Amino acid transporters (*AATs*) are a kind of membrane proteins that mediate the transport of amino acids across cell membranes in higher plants. The AAT proteins are involved in regulating plant cell growth and various developmental processes. However, the biological function of this gene family in cotton fiber development is not clear. In this study, 190, 190, 101, and 94 full-length *AAT* genes were identified from *Gossypium* *hirsutum*, *G. barbadense*, *G. arboreum*, and *G. raimondii*. A total of 575 *AAT* genes from the four cotton species were divided into two subfamilies and 12 clades based on phylogenetic analysis. The *AAT* genes in the four cotton species were distributed on all the chromosomes. All *GhAAT* genes contain multiple exons, and each GhAAT protein has multiple conserved motifs. Transcriptional profiling and RT qPCR analysis showed that four *GhATT* genes tend to express specifically at the fiber initiation stage. Eight genes tend to express specifically at the fiber elongation and maturity stage, and four genes tend to express specifically at the fiber initiation and elongation stages. Our results provide a solid basis for further elucidating the biological function of *AAT* genes related to cotton fiber development and offer valuable genetic resources for crop improvement in the future.

## 1. Introduction

Amino acids are essential in the growth and development of plants. Plants can absorb amino acids from the soil (exogenous amino acid) or synthesize amino acids themselves (endogenous amino acid). Amino acids can be absorbed and utilized directly by plant roots and transported to root tips, flowers, growing leaves, fruits, and seeds through xylem and phloem to sustain plant growth and development. The process of amino acid transport usually requires the participation of amino acid transporters [1,2].

Amino acid transporters (*AAT*s) are a kind of membrane proteins that mediate the transport of amino acids across the cell membrane in higher plants and play an indispensable role in all processes of plant growth and development. *AAT*s participate in long-distance amino acid transport, response to pathogens and abiotic stress, etc., [3,4,5,6,7,8,9]. According to the sequence similarity of AAT proteins, the *AAT* family in plants mainly includes the amino acid polyamine choline transporter (APC) subfamily and the amino acid/auxin permease (AAAP) subfamily—also known as the amino acid transporter (ATF) subfamily. Subsequently, the APC subfamily is divided into three groups, including cationic amino acid transporters (CATs), amino acid/choline transporters (ACTs), and polyamine H+ symporters (PHSs). The AAAP subfamily contains at least eight groups, including amino acid permeases (AAPs), lysine and histidine transporters (LHTs), γ-aminobutyric acid transporters (GATs), proline transporters (ProTs), auxin transporters (AUXs), amino acid transporters-like (ATLs), aromatic and neutral amino acid transporters (ANTs), and tyrosine-specific transporters (TTPs) [9,10,11,12,13]. The *AAT* gene family has been systematically identified and analyzed in several model or crop species. At least 189 *AAT* genes have been identified as members of the *AAT* family in soybean (*Glycine max* L.) [14], 296 genes in wheat (*Triticum aestivum* L.) [9], 85 genes in rice (*Oryza sativa* L.) [15], 72 genes in potato (*Solanum tuberosum* L.) [2], and 107 genes in maize (*Zea mays* L.) [16]. Although the *AAT* family had been identified in various plants, the most detailed study of its function has been carried out in *Arabidopsis thaliana*.

As far as the AAAP subfamily is concerned, eight AAPs have been found in *A. thaliana*, with 9–10 transmembrane regions. Except for *AtAAP7*, which is in the chloroplast vesicle membrane, others are in the plasma membrane [5]. Fischer and others first discovered *AAP* gene in *A. thaliana* in 1995 through amino acid deletion and absorption experiments in yeast strains [17]. *AtAAP1* is expressed in roots and seeds, transports glutamic acid, histidine, and neutral amino acids in soil, and regulates endosperm absorption of amino acids [18,19]. *AtAAP2* is expressed in phloem and transports glutamic acid (Glu) and neutral amino acids from the xylem to phloem [17,20,21]; *AtAAP3* is mainly expressed in vascular bundles of roots, which may have the function of absorbing neutral and basic amino acids from phloem or transporting amino acids from soil [20]; AtAAP4 is expressed in leaves, stems and flowers and transports neutral amino acids proline (Pro) and valine (Val) [17]. *AtAAP5* is expressed in all tissues of *A. thaliana* and plays an essential role in absorbing anionic, neutral, and cationic amino acids in roots [21]; *AtAAP6* is mainly expressed in xylem and transports tryptophan, proline, and neutral and acidic amino acids [17,22]; *AtAAP8* is expressed in seeds, and the mutation of this gene can reduce the seed setting rate of *A. thaliana* by about 50% [23]. Like the function of AAPs in *A. thaliana*, AAPs have also been studied in rice, wheat, maize, poplar, pea, broad bean, and other plants, all of which show that AAPs play an essential role in plant growth and development [2,24,25]. There are 10 LHT members in *A. thaliana*, all of which are located in the plasma membrane. However, only two LHT members have been characterized; *AtLHT1* is highly expressed in young leaves, flowers, and pods. *AtLHT1* transports neutral and acidic amino acid [26]. *AtLHT6* is mainly expressed in root and transports acidic amino acids, glutamine, and alanine [27]. ProTs usually transport glycine, betaine, imino proline, amino acid a-aminobutyric acid (GABA), and quaternary ammonium compounds. There are three ProTs members in *A. thaliana*, all of which are located in the plasma membrane. *AtProT1* is expressed in phloem or phloem parenchyma cells, which proves that the protein plays a role in the long-distance transport of amino acids. *AtProT2* is expressed in roots and transports proline and glycine betaine. *AtProT3* is expressed in the leaf epidermis, and its protein is involved in the transport of proline [8,22,28,29,30]. There are two GAT members in *A. thaliana*. *AtGAT1*, a transporter of GABA, ω-amino fatty acids, and butylamine, is highly expressed in flowers [31]. ANTs are a new class of amino acid transporters. There are four ANT members in *A. thaliana*, which are expressed in all organs. In addition to aromatic and neutral amino acids and arginine, the protein can also transport indole-3-acetic acid and 2,4-dichlorophenoxyacetic acid [26].

There are 17 members of the APC family in *A. thaliana*, of which nine belong to the CAT clade, five to the PHS clade, and one to the ACT clade. The CAT clade consists of nine members with high sequence similarity, mainly located on the plasma membrane and tonoplast, with 11–14 transmembrane domains [32]. *AtCAT1* can promote the high-throughput absorption of essential amino acids and belongs to a specific nitrogen-containing metabolite transport gene, which is less expressed in roots and highly expressed in leaves, flowers, and pods up-regulated rapidly after infection with pseudomonas lanceolate. It is inferred that it may be involved in the systematic response to the attack of pathogens in plants [7,33]. *AtCAT2*, *AtCAT4*, and *AtCAT9* are all located in the vesicular membrane, and *AtCAT2* is related to the concentration of amino acids in leaves. It is speculated that *AtCAT2* is associated with the transport of amino acids through the tonoplast [7]. *AtCAT5* is expressed in seeds. It is supposed that the gene may play a role in seed germination or seedling development by transporting essential amino acids from peduncle to seeds. *AtCAT3* is located on the endoplasmic reticulum and transports cationic, neutral, or acidic amino acids. *AtCAT6* is expressed in roots and transports cationic neutral or acidic amino acids. The expression of *AtCAT8* is high in stem tip meristem and root tip meristem and transports cationic neutral or acidic amino acids [6].

Cotton is an important cash crop that can produce natural fiber and be used in textile [34,35]. The yield and quality of cotton fiber are the most critical factors determining the economic value of cotton [36]. Cotton has two categories: cultivated species and wild species. In taxonomy, cotton belongs to the *Gossypium* genus [37]. There are 4 subgenus and 52 species in the genus *Gossypium* L., including 45 diploid cotton species (2*n* = 2*x* = 26) with eight genomic types, A, B, C, D, E, F, G, and K, and 7 allotetraploid cotton species (2*n* = 4*x* = 52). The genome types of allotetraploid cotton species are AD. The AD genome is formed by interspecies hybridizing the genome A and the genome D [36,38].

Evolutionary analysis showed that the D subgenome of allotetraploid cotton species originated from the D5 genome of *G.raimondii* L. The A subgenome of allotetraploid cotton species, A1 genome of *G. herbaceum* L., and A2 genome of *G. arboreum* L. were all originated from the A0 genome of extinct cotton [39].

There are four cultivated types of cotton: *G. herbaceum* L. (A1), *G. arboreum* L. (A2), *G. hirsutum* L. ((AD) 1), and *G. barbadense* L. ((AD) 2). *G. hirsutum* L. is widely popularized because of its high yield and strong adaptability, accounting for more than 95% of the cultivated cotton area. *G. barbadense* L. has good fiber quality and low yield. It can only be planted in a few arid areas [39,40]. There is interspecific reproductive isolation between *G. hirsutum* L. and *G. barbadense* L. How to combine their advantages and cultivate cotton varieties with high yield, high quality, and wide adaptability has always been a research hotspot [41,42].

Cotton fiber cell is a single-celled trichome formed by the differentiation and growth of ovule epidermal cells. It is the non-branching single cell with the fastest growth rate and the longest development time in higher plants, and it is also the best model for the study of single cells [43,44]. The development of cotton fiber consists of five overlapping stages: fiber initiation (−3 DPA–5 DPA), elongation (5–16 DPA), transition (16–20 DPA), secondary cell wall biosynthesis (20–40 DPA), and maturation (40–50 DPA) [45,46,47]. There are two types of cotton fibers, long fiber (2.5 to 3.5 cm in length) and adherent fuzz fiber (5 to 10 mm in length). Long fiber is the essential raw material in the textile industry, which begins to develop on the day of anthesis (0 DPA). The fiber initiation stage of cotton is the critical stage determining cotton yield. Because this stage determines how many ovule epidermal cells develop into fibers. About 25% to 30% of the ovule epidermis cells eventually differentiate into cotton fibers [47,48]. The elongation, transition, secondary cell wall biosynthesis are the key stages determining fiber development and quality [46]. The analysis of the molecular mechanism of cotton fiber development will provide an essential theoretical basis for increasing cotton yield and improving cotton fiber quality. The publication of *G. raimondii* L., *G. arboreum* L., *G. hirsutum* L., *G. herbaceum* L., and *G.* *barbadense* L. genomes [40,48,49,50,51,52] will provide an essential theoretical basis for our comprehensive analysis of specific gene families.

The *AAT* gene family has been systematically identified in many plants [53,54,55]. Due to the complex genomic structure of cotton, little is known about *AAT* genes in cotton. Here, we analyzed the whole genome of the cotton *AAT* gene family. We identified amino acid transport protein (*AAT*) genes in four cotton species: the tetraploid *G. hirsutum* L. and *G. barbadense* L., which had 190 and 190 *AAT* genes respectively. Their putative extent parental diploids *G. arboreum* L. and *G. raimondii* L. had 101 and 104 AATs, respectively.

To explore the functions of *AAT* genes in cotton fiber development, we analyzed the expression profiles of *GhAAT* genes during cotton fiber development. We found that some *AAT* genes play an important role in fiber development. The findings from this study will lay a foundation to understand the genomic organization and functional structure of the *AAT* gene family in the available genomes of four species of cotton that will be useful in characterization of the functional genomics.

## 2. Materials and Methods

### 2.1. Identification of AAT Genes in Cotton

The four genome files of *G. arboreum* (CRI, version 1.0) [50], *G. raimondii* (JGI, version 2.0) [49], *G. hirsutum* (HAU, version 1.1) [42,52,56], and *G. barbadense* (ZJU, version 1.1) [41] were downloaded from the Cotton Functional Genomics Database (CottonFGD) (https://cottonfgd.org/, accessed on 10 July 2021) [56]. The genome sequences of *A. thaliana* were retrieved from JGI (https://phytozome.jgi.doe.gov/pz/portal.html, accessed on 10 July 2021). The Hidden Markov Model (HMM) profile of AA_trans (PF01490) and AA_permease (PF00324) were downloaded from Pfam (https://pfam.xfam.org, accessed on 10 July 2021). Then we used HMMER 3.0 software (http://www.hmmer.org/, accessed on 10 July 2021) with an e-value of 1 × 10^−5^ as the threshold to acquire the AAT protein sequences who including PF01490 or PF00324, which are most probably members of the *AAT* gene family. All putative AATs were confirmed by motif scanning on Pfam (https://pfam.xfam.org, accessed on 10 July 2021), and the genes contained AA_trans domain or AA_permease domain was employed for further analysis. The transmembrane domain was predicted using TMHMM Server v2.0 (http://www.cbs.dtu.dk/services/TMHMM/, accessed on 10 July 2021). We retrieved some biochemical parameters of AAT proteins in *G. hirsutum* L., such as isoelectric points (pIs), molecular weights (MWs), Grand Average of Hydropathy, and charge by using Cotton Functional Genomic Database (CottonFGD) (https://cottonfgd.org/, accessed on 10 July 2021) [56]. We also retrieved the exons and introns structures of *AAT* genes in *G. hirsutum* L. by using Cotton Functional Genomic Database (CottonFGD) (https://cottonfgd.org/, accessed on 10 July 2021) [56]. For predicting the subcellular location predict of GhAAT proteins, we used the website WOLF-PSORT (https://wolfpsort.hgc.jp/, accessed on 10 July 2021) [57].

### 2.2. Sequence Alignment and Phylogenetic Analysis

The full-length amino acid sequence of *G. hirsutum*, *G. arboreum*, *G. raimondii*, *G. barbadense*, and *A. thaliana* encoded by *AAT* genes were aligned using the online ClustalW program (https://www.genome.jp/tools-bin/clustalw, accessed on 10 July 2021) with default settings. We constructed the maximum likelihood (ML) trees with 1000 bootstrap replicates using the Poisson substitution model with default parameters in MEGAX [58], and the trees were modified by the EvolVeiw (https://www.evolgenius.info/evolview, accessed on 10 July 2021) [59].

### 2.3. Chromosomal Locations of AATs from Four G. Species

Genomic sequences, CDS sequences, and GFF (general feature format) information of all four species were downloaded from Cotton Functional Genomic Database (CottonFGD) (https://cottonfgd.org/, accessed on 10 July 2021) [56]. The physical chromosome locations of all *AATs* members were visualized by TBtools software [60].

### 2.4. Gene Duplication and Synteny Analysis in Different G. Species

The synteny analysis between duplicated gene pairs from four cotton species, *G. hirsutum* L., *G. arboreum* L., *G. raimondii* L., and *G. barbadense* L., was analyzed by using MCScanX software and JCVI [61]. The results were drawn by simple Circos software (http://circos.ca/, accessed on 10 July 2021) [62].

### 2.5. Calculation of Selection Pressure

The homologous gene pairs of four cotton species (*G. arboreum* L., *G. raimondii* L., *G. hirsutum* L., and *G. barbadense* L.) were identified by MCSCanX [61]. We calculated the non-synonymous to the synonymous mutations rate (Ka/Ks) of homologous genes to examine the selection pressure. Selection pressure analysis was performed by calculation of the Ka (non-synonymous substitution rate) and Ks (synonymous substitution rate) values of repetitive genes using KaKs_Calculator 2.0 software [63].

### 2.6. Analysis of the Conserved Protein Motifs and Gene Structure

The Gene Structure Display Server (GSDS) online tool (http://gsds.gao-lab.org/, accessed on 10 July 2021) [64] was used to visualize the structures, including genomic sequences and positions of exons and introns of *GhAATs*. The Multiple EM for Motif Elicitation (MEME) program [65] was used to analyze the conserved protein motifs of the whole protein sequences of GhAATs. The parameters of which were as follows: the optimal motif width was between 6 and 200 residues, the maximum number of motifs was set to 20, the motif number distributed in sequences was set to 0 or 1, and the remainder of the parameters were set to system defaults. The phylogenetic tree figure, along with gene structure and conserved protein motifs, was drawn with TBTools software [60] using MAST file from MEME website, NWK file from phylogenetic tree analysis, and GFF3 genome file of *G. hirsutum* L.

### 2.7. Analysis of AATs Promoter Regions

The 5′ untranslated regions 2000 bp sequence upstream of the transcription start site (TTS) in the genomic DNA sequence of *GhAATs* as promoters were extracted from the CottonFGD database (https://cottonfgd.org/, accessed on 10 July 2021) [56]. The online software HOMER (http://homer.ucsd.edu/homer/, accessed on 10 July 2021) was used to predict Cis-acting elements in promotor regions of *GhAAT* genes [66].

### 2.8. Gene Ontology (GO) Annotation/Gene Enrichment Analysis

We used the Cotton functional genomic database (CottonFGD) (https://cottonfgd.org, accessed on 10 July 2021) to determine the functional classification of *GhAATs* genes, including biological process, cellular, and molecular functions [56].

### 2.9. Gene Expression Pattern Analysis

We collected the ovules of *G. hirsutum* L. TM-1 in the following six stages: −3 DPA, −1 DPA, 0 DPA, 1 DPA, 3 DPA, and 5 DPA, and three biological repeats were collected in each stage. We also collected the fibers of *G. hirsutum* L. in the following five stages: 7 DPA, 10 DPA, 15 DPA, 20 DPA, and 30 DPA, and three biological repeats were collected in each stage. All samples were immediately frozen in liquid nitrogen and stored at −80 °C [47]. Total RNA was extracted by the Total RNA Extraction Kit (R1200) (Beijing Solarbio Science & Technology Co., Ltd., Beijing, China) from all the samples. The Goldenstar™ RT6 cDNA Synthesis Kit (Beijing Tskingke Biotech Co., Ltd., Beijing, China) was used to reverse the extracted RNA to obtain the first-strand cDNA for transcriptomic analysis. cDNA libraries were constructed and subjected to 101-cycle paired-end sequencing on an Illumina HiSeq 4000 platform at Berry Genomics (Beijing, China). The data were normalized using the reads per kilobase of exon model per million mapped reads (FPKM) algorithm. The FPKM value of all genes in *G. hirsutum* L. cultivar Texas Marker-1 (TM-1) during fiber development is shown in Table S26. The *GhAATs* with an FPKM > 1 or log2 (1 + FPKM) > 1 at least in one fiber development stage were thought to be expressed in ovules and fibers and were employed for further analysis. The genes were divided into specific and nonspecific expressions using the formula in previous study [38]. According to this formula, we wrote a script using Python for expression and tissue specificity analysis (Additional file1). The expression heatmaps were visualized by TBtools [60].

### 2.10. RNA Extraction and RT qPCR Analysis

Plant total RNA was extracted by the Total RNA Extraction Kit (R1200) (Beijing Solarbio Science & Technology Co., Ltd., Beijing, China). The reverse transcription kit was the PrimeScript™ II 1st Strand cDNA Synthesis Kit (TAKARA, Dalian, China). It was used to reverse the extracted RNA to obtain the first-strand cDNA for transcriptomic and RT qPCR analysis. The expression heatmap was visualized by Tbtools [60]. The fluorescent quantitative kit was the Taq Pro Universal SYBR qPCR Master Mix (Q712-02) (Vazyme Biotech Co., Ltd, Nanjing China). The data were calculated according to the 2^−ΔΔ CT^ method. The *G. hirsutum* L. the *His3* (*GhHis3*) gene were used as reference controls [48]. According to the candidate gene sequence, a relatively specific primer for real-time fluorescence quantitative PCR was designed by Primer3 software, and the amplification product was 150–300 bp. The RTqPCR primers used in this study are shown in Table S23. All primers were synthesized by (Sangon Biotech Co., Ltd, Shanghai, China).

## 3. Results

### 3.1. Genome Wide Identification of AATs in G. raimondii L., G. arboreum L., G. hirsutum L., and G. barbadense L.

In this study, we identified 190, 190, 101, and 94 full-length amino acid transporter (*AAT*) genes from *G. hirsutum* L. (allotetraploid cotton), *G. barbadense* L. (allotetraploid cotton), *G. arboreum* L. (diploid cotton), and *G. raimondii* L. (diploid cotton) respectively. It was confirmed that the AAAP subfamily protein contained an AAT-trans domain, and the APC subfamily protein sequence contained one or more AA_permease or AA_permease_2. Besides, the CAT group belonged to the APC subfamily and specifically has an AA_permease_C characteristic domain.

The identified *AAT*s members of the four cotton species were named according to their chromosomal locations. To facilitate comparison, the *GhAATs* are also named according to their evolutionary relationship with *A. thaliana*. Gh, Gb, Ga, Gr, and At were used as prefixes before the names of *AAT* genes from *G. hirsutum* L., *G. barbadense* L., *G. arboreum* L., *G. raimondii* L., and *A. thaliana*, respectively. The detailed results are shown in Tables S1–S5.

Subsequently, we determined the features of the *AATs* members of the *G. hirsutum* L. including genomic length (bp), protein length (aa), CDS length (bp), locus ID with corresponding chromosome number, strand polarity, start and end points, predicted isoelectric points (PI), predicted masses and protein molecular weights (MW) which were shown in Table S1. The protein length of *GhAAT* genes ranged from 139 (*GhAAT*111, localized on Ghir_D09 chromosome) to 980 (*GhLAT15*, localized on Ghir_D12 chromosome) amino acids (aa). The isoelectric point (PI) of *GhAAT* genes varied from 4.75 (*GhAtLb8*) to 9.66 (*GhLHT13*). The numbers of TM (transmembrane) in *GhAAT* family members ranged from 3 (*GhAAP25*) to 14 (*GhAAPL2*). We found all the *GhAAT* proteins were located in the plasma membrane (Table S1).

### 3.2. Sequence Alignment and Phylogenetic Analysis

To examine the evolutionary relationships of *AAT* genes in *G. hirsutum* L., phylogenetic analysis of all 190 GhAAT protein sequences was performed to construct the unrooted tree based on multiple sequence alignment, using the maximum likelihood (ML) method in MEGA X. Based on previous *AAT* family study in other organisms, we directly divided *Gh*AAT members into two main subfamilies—AAAP subfamily and APC subfamily. The AAAP subfamily could be further divided into ten groups, including AAP, LHT, GAT, ProT, AUX, ATLa, ANT, ATLb, AAPL, and TTP. The APC subfamily was composed mainly of three groups—PHS, ACT, and CAT groups, respectively. The AAAP and the APC subfamily contained 143 and 47 members, respectively. In the AAAP subfamily, we discovered that AAP was the largest group, including 28 AAT members. AAPL and TTP were the smallest groups, with only two AAT members each. The other groups contained 24 (LHT), 10 (GAT), 6 (ProT), 17 (AUX), 22 (ATLa), 10 (ANT), and 22 (ATL). In the APC family, we discovered that CAT was the largest group, including 28 AAT members, ACT was the smallest group with only four AAT members, and PHS contained 15 members (Figure S1 and Table S1).

**Figure 1:**
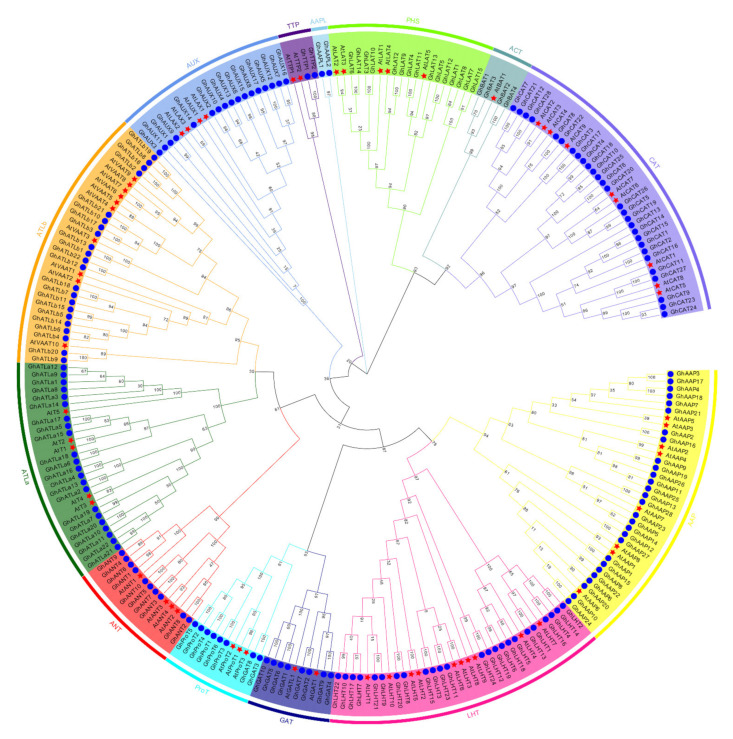
Phylogenetic tree of GhAAT (*G. hirsutum* L. AAT) and AtAAT (*A. thaliana* AAT) proteins. 190 GhAATs from *G.* *hirsutum* L. marked by red pentagrams and 63 AtAATs from A. thaliana marked by blue cycle. The phylogenetic tree was generated using MEGAX via the maximum likelihood (ML) method with 1000 bootstrap replicates. All 190 GhAATs and 63 AtAATs were divided into 13 subgroups which were highlighted by different colors (AAP, LHT, GAT, ProT, AUX, ATLa, ANT, ATLb, AAPL, TTP, PHS, ACT, and CAT).

*A. thaliana* contained 63 AATs (AtAATs) members. To examine the evolutionary and orthologous relationships of *AAT* genes between *G. hirsutum* L. and *A. thaliana*, 63 AtAATs and 190 GhAATs protein sequences were performed to construct the phylogenetic tree, using the maximum likelihood (ML) method in MEGA X, which were also divided into 13 groups (Figure 1). However, all 63 AtAATs were distributed in all groups except group AAPL, specific for *G. hirsutum* L., containing only two GhAATs—GhAAPL1 and GhAAPL2. Interestingly, when the AAT proteins of *G. hirsutum* L. and *A. thaliana* were used to construct the phylogenetic tree, two genes *GhGAT3* and *GhGAT8*, which initially belonged to the GAT group, were divided into the ProT group, which indicated that GAT and ProT might evolve in the same direction, and *GhGAT3* and *GhGAT8* had homology with *A. thaliana* and cotton ProT group genes (Figure 1).

To study the evolutionary relationship between allotetraploid *G. hirsutum* L. and *G. barbadense* L. and diploid cotton *G. arboreum* L. and *G. raimondii* L., we used the same method to construct a phylogenetic tree based on the protein sequences of AAT families of four cotton species. The results showed that all cotton AAT family proteins were divided into 12 clearly defined clades, almost consistent with the above two evolutionary trees, each clade contained family genes from diploid and allotetraploid cotton species. The AAPL group that existed separately in the *G. hirsutum* L. phylogenetic tree was divided into the AAP family in the phylogenetic tree of four cotton species. It is suggested that the two AAPL family genes may have similar functions to AAP group genes (Figure S2).

### 3.3. Chromosomal Locations of AATs from Four Gossypium Species

**Figure 2:**
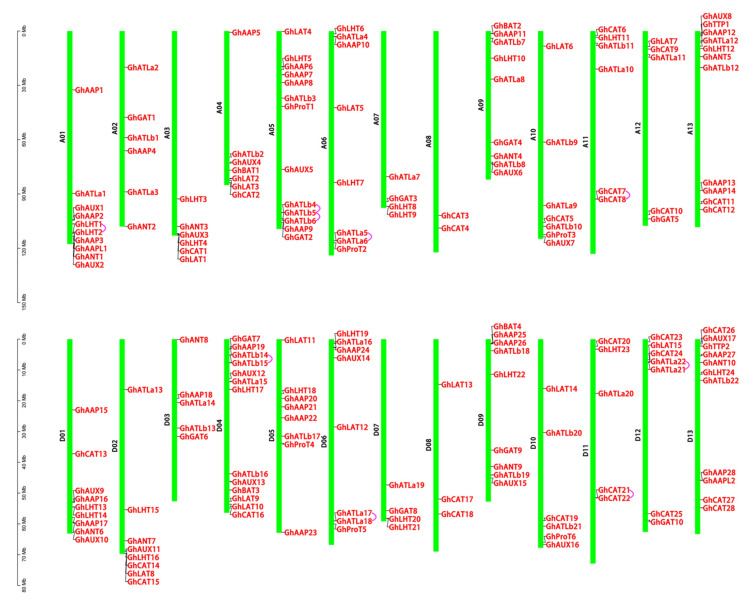
Chromosomal distribution of *AATs* in *G. hirsutum* L. The scale represented megabases (Mb). The chromosome IDs were indicated beside each vertical bar. The *AATs* were displayed on different chromosomes. Green bars represented the physical maps of chromosomes in *G. hirsutum* L. and the purple short arc linked genes were the tandem repeat genes in GhAATs.

To better understand the distribution mechanism of *AAT* family genes on cotton chromosomes, genomes, and subgenomes and analyze their structure, we mapped the chromosomal maps of 575 *AAT* members of two allotetraploid cotton species, *G. hirsutum* L. and *G. barbadense* L., and two diploid cotton species, *G. raimondii* L. and *G. arboreum* L. Among the 190 *AAT* identified in *G. hirsutum* L., 94 genes were located on 13 chromosomes of GhAt (A subgenome of *G. hirsutum* L. genome), and the remaining 96 genes were found on 13 chromosomes of GhDt (D subgenome of *G. hirsutum* L. genome) (Figure 2 and Table S6). The number of *GhAATs* on GhAt and GhDt was close to 1:1. The number and distribution of *GhAATs* on homologous chromosomes of different subgenomes were highly similar. GhAt05 (13) and GhDt04 (13) had the most number of *AAT* genes, while GhAt08 (2) and GhDt08 (3) had the least number of *AAT* genes, and there are 3 to 11 on other chromosomes (Figure 2). Besides, except for (GhAt06-GhDt06), (GhAt07-GhDt07), and (GhAt13-GhDt13), the number of genes on the chromosome of subgenome A is different from that of the homologous chromosome of subgenome D (Figure 2 and Table S6). The *AAT* gene numbers distributed on each chromosome of Gossypiums are showed in Table S6.

Among the 190 *AAT* genes of *G. barbadense* L., 189 locates on 26 chromosomes, and the other one locates on a scaffold (Figure S3 and Table S6). GbAt (A subgenome of *G. barbadesnse* genome) contains 96 genes, GbDt (D subgenome of *G. barbadesnse* genome) contains 93 *AATs*. Like *G. hirsutum* L., the number of *GbAATs* on GbAt and GbDt was close to 1:1, and the number and distribution of *GbAATs* on homologous chromosomes of different subgenomes were highly similar. The number of genes on GbAt05 was the most, with 14 *GbAAT* members, and the number of genes on GbAt07, GbDt07, and GbDt08 was the least, with only three genes (Figure S3 and Table S6). Of the 101 identified *AAT* genes in *G. arboreum* L., 100 were mapped to 13 chromosomes, and only one *AAT* was located on a scaffold (Figure S4 and Table S6). All 94 *AAT* genes of *G. raimondii* L. were unevenly distributed on 13 chromosomes (Figure S5 and Table S6). The number of D12 and D13 genes was the most, with 13 genes, while D01, D03, and D04 genes were the least, with only three genes. The A and D subgenomes of allotetraploid *G. hirsutum* L. and *G. barbadense* L. were compared with their diploid *G. arboreum* L. and *G. raimondii* L., respectively. It was found that the number and distribution of genes on the chromosomes of two tetraploid cotton A and D subgenomes were almost the same as those of diploid cotton A and D genomes.

### 3.4. Gene Duplication and Synteny Analysis in Different Gossypium Species

The gene family’s evolution has experienced three processes: whole genome duplication or polyploidization, segmental duplication, and tandem duplication. They provide the main forces driving the formation of gene families and their evolution [9]. Based on the open reading frame (ORF) sequence of all genes in each species, the *AAT* family genes of *G. hirsutum* L., *G. barbadense* L., *G. arboreum* L., and *G. raimondii* L. were analyzed to determine the gene duplication and collinear relationship.

**Figure 3:**
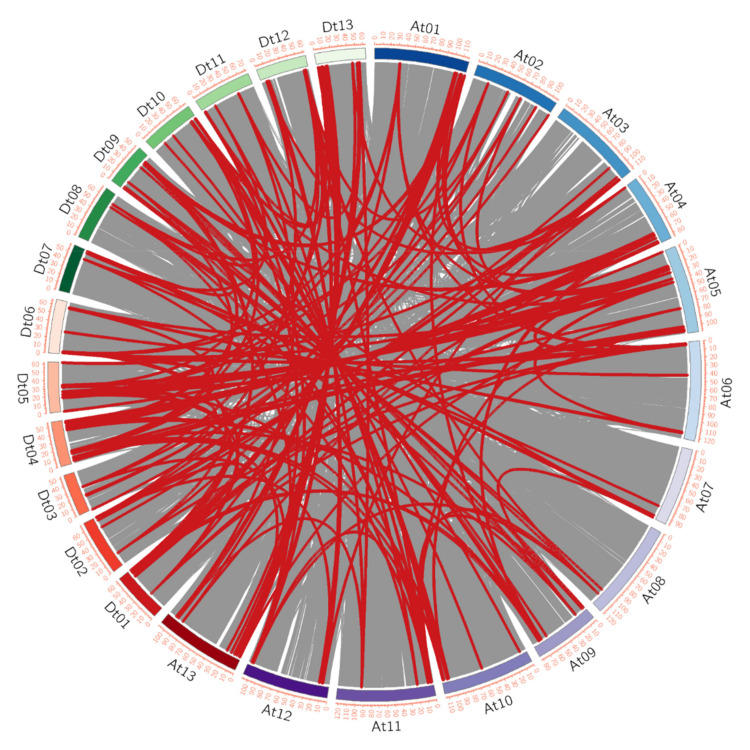
The syntenic relationship of *GhAAT*. The synteny of *GhAATs* in *G. hirsutum* L. marked with red lines. The grey lines indicated all the synteny relationships in *G. hirsutum* L. genome.

**Figure 4:**
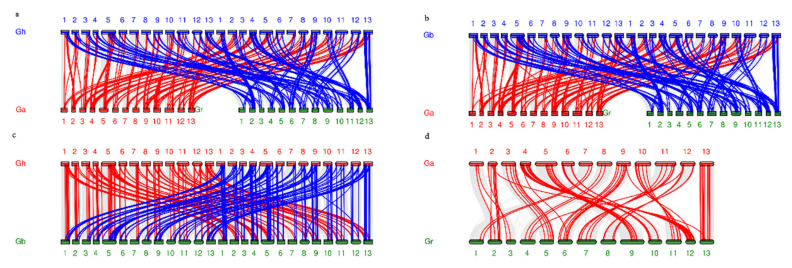
The syntenic relationship between the *AATs* of four *G.* species. (**a**) The synteny of *GhAATs* between *G. hirsutum* L. and *G. arboreum* L. labeled with red lines and the synteny of *GhAATs* between *G. hirsutum* L. and *G. raimondii* L. labeled with blue lines. The grey lines indicated all the syntenic relationships in three *G.* genomes. (**b**) The synteny of *GbAATs* between *G. barbadense* L. and *G. arboreum* L. labeled with red lines and the synteny of *GbAATs* between *G. barbadense* L. and *G. raimondii* L. labeled with blue lines. The grey lines indicated all the syntenic relationships in three *G.* genomes. (**c**) The synteny of *GhAATs* between *G. hirsutum* L. and *G. barbadense* L. labeled with red lines and the synteny of *GbAATs* between *G. barbadense* L. and *G. hirsutum* L. labeled with blue lines. The grey lines indicated all the syntenic relationships in two *G.* genomes. (**d**) The synteny of *GaAATs* between *G. arboreum* L. and *G. raimondii* L. labeled with red lines.

A total of 202, 216, 32, and 41 gene-pairs with segmental duplication were discovered in *G. hirsutum* L., *G. barbadense* L., *G. arboretum,* and *G. raimondii,* respectively (Figure 3 and Tables S7, S9, S11 and S13). As well, we identified 9, 5, 2, and 5 tandem duplicated gene-pairs in corresponding species (Tables S8, S10, S12 and S14). All these results indicated that both segmental and tandem duplication played essential roles in the expansion of *AAT* family proteins in *G. hirsutum* L., *G. barbadense* L., *G. arboreum* L., and *G. raimondii* L., while segmental duplication were predominantly present in four *Gossipium* species. In addition, the distribution of genome-wide duplication gene pairs between four Gossipium species is displayed in the Figure 4 and Tables S15–S21.

### 3.5. Selective Pressure Analysis of AAT Genes in Cotton

Calculating the replacement rates of non-synonymous (Ka) and synonymous (Ks) is an effective method to evaluate the homologous sequence variation of proteins in different species or classifications with unknown evolutionary status [32]. The value of Ka/Ks represents the ratio of Ka and Ks of two homologous protein-coding genes. Ka/Ks > 1 indicates positive selection, while Ka/Ks = 1 indicates neutral selection, and Ka/Ks < 1 indicates purifying or stabilizing selection. The Ka/Ks value of one pair of *GhAAT* paralogous genes in *G. hirsutum* L. was greater than one, accounting for 0.47% of them (1/211), the Ka/Ks values of the rest of them were in the range of 0 and 0.79. The Ka/Ks values of 9 pairs of *AAT* orthologous genes between allotetraploid *G. hirsutum* L. and *G. barbadense* L. were greater than 1, accounting for 3.05% (9/295), the Ka/Ks value of one pair of them was greater than two, accounting for 0.34% of them (1/295), and the Ka/Ks values of the rest of them were in the range of 0 and 0.99. The Ka/Ks values of 5 pairs of *AAT* orthologous genes between allotetraploid *G. hirsutum* L. and diploid *G. arboreum* L. were greater than one, accounting for 3.47% of them (5/144), and the Ka/Ks values of the rest of them were in the range of 0 and 0.97. The Ka/Ks values of four pairs of *AAT* orthologous genes between allotetraploid *G. hirsutum* L. and diploid *G. raimondii* L. were greater than one, accounting for 2.65% of them (4/151), the Ka/Ks values of two pairs of them were greater than 2, accounting for 1.32% of them (2/151), and the rest were in the range of 0 and 0.98. The Ka/Ks values of 8 pairs of *AAT* orthologous genes between allotetraploid *G. barbadense* L. and diploid *G. arboreum* L. were greater than one, accounting for 5.23% of them (8/153), and the Ka/Ks values of the rest of them were in the range of 0 and 0.97. The Ka/Ks values of 6 pairs of *AAT* orthologous genes between allotetraploid *G. barbadense* L. and diploid *G. raimondii* L. were greater than one, accounting for 3.53% of them (6/170), the Ka/Ks values of two pairs of them were great than 2, accounting for 1.18% of them (2/170), and the Ka/Ks values of the rest of them were in the range of 0 and 0.95. The Ka/Ks value of one pair of *AAT* paralogous genes in allotetraploid *G. barbadense* L. was greater than 1, accounting for 0.45% of them (1/221), and the Ka/Ks values of the rest of them were in the range of 0.02 and 0.88. The Ka/Ks values of *AAT* orthologous genes between diploid cotton species *G. arboreum* L. and *G. raimondii* L. were in the range of 0.01 to 0.92. The Ka/Ks values of *AAT* paralogous genes in *G. arboreum* L. were in the range of 0.02 and 0.37. The Ka/Ks values of *AAT* paralogous genes in *G. raimondii* L. ranged from 0.02 to 0.35 (Table S25).

### 3.6. Analysis of the Conserved Protein Motifs and Gene Structure

**Figure 5:**
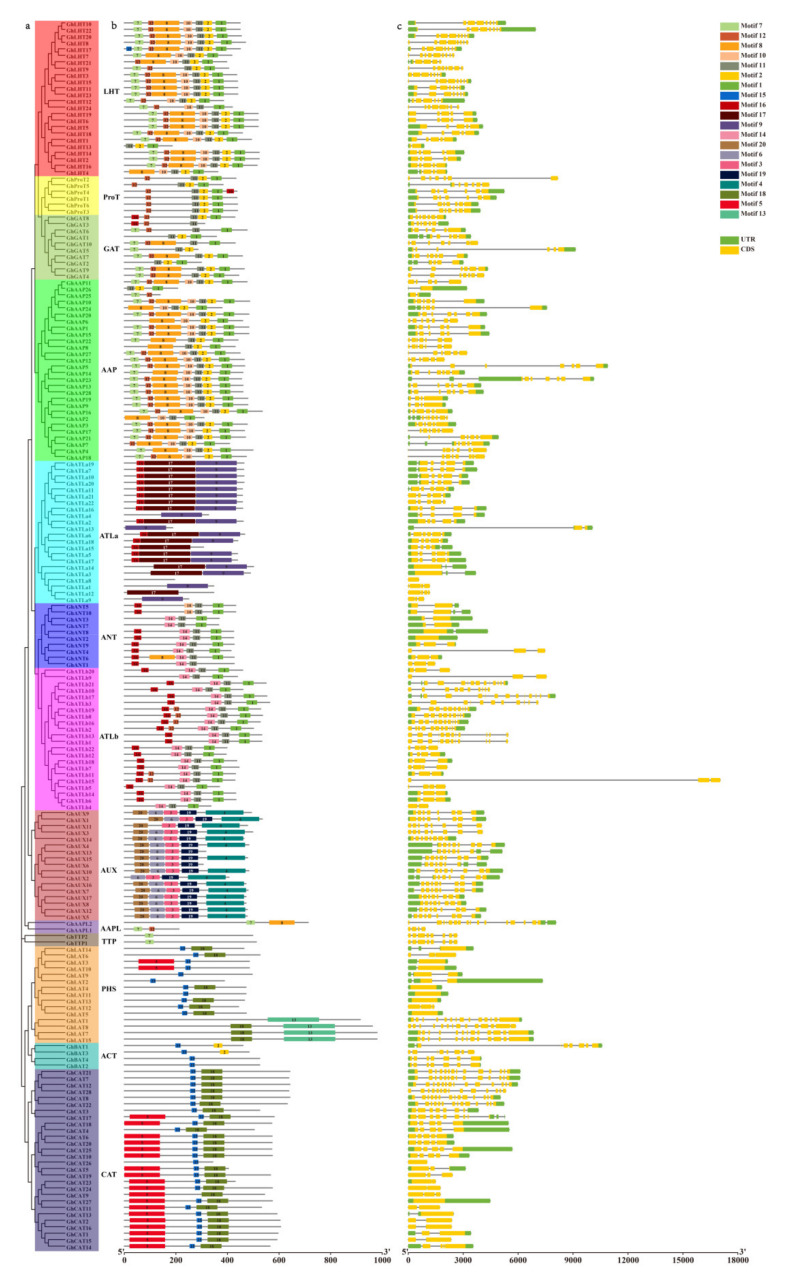
Phylogenetic relationships, protein domains, and gene structure analysis of *GhAATs*. (**a**) Phylogenetic analysis of GhAATs proteins using MEGAX via the maximum likelihood (ML) method. (**b**) The conserved protein motifs of the amino acid sequences of GhAATs were analyzed by the MEME. (**c**) The gene structures of *GhAATs*. Gene structure analysis was performed by Gene Structure Display Server (GSDS). The CDSs, untranscipted regions (UTRs) and introns are indicated with yellow rectangles, blue rectangles, and black lines, respectively.

During the evolution of gene families, the diversification of protein domain is responsible for the evolution of new protein function to acclimatize in the changing environment. Therefore, the protein domain gene structure analysis was performed to identify the conserved motifs present in GhAATs. Twenty possible motifs were identified in GhAAT members by the Meme website. These motifs were regularly distributed in the 190 GhAATs. These motifs are variable among clades but remain conservative in clades. LHT, GAT, ProT, and AAAP clades were adjacent to each other in the evolutionary tree, and their motif composition was similar. However, compared with LHT, GAT, and AAAP clades, ProT clade lacked motif 7, motif 8, and motif 10. ATLa, ANT, and ATLb clades were in adjacent positions in the evolutionary tree. Compared with other clades of the AAT family, motif 16 existed explicitly in these three clades. The motif composition of ANT and ATLb clades was very similar. Compared with other clades of the AAT family, motif 9 and motif 19 were specific in the ATLa clade. Compared with other clades of the AAT family, motif 3, motif 4, motif 6, motif 19, and motif 20 existed specifically in the AUX clade. The TTP clade contained only one motif—motif 7. Motif 15 and motif 18 were ubiquitous in the three clades of the APC clade (PHS, ACT, CAT). Unlike other clades of the AAT family, motif 13 existed specifically in the PHS clade, motif 2 existed specifically in the ACT clade, and motif 5 existed specifically in the CAT clade (Figure 5b).

Furthermore, we explored the structural diversity of *GhAAT* genes. The exon-intron distribution pattern of the gene is related to the phylogeny of genes [67,68,69]. Gene structure analysis showed consistent results to our phylogenetic analysis. The exon number of *GhAAT* genes ranged from 1 to 12. The number of exons is different among different clades, and the exon-intron composition of most *GhAAT* members in the same clade was similar (Figure 5c and Table S1).

### 3.7. Gene Expression Pattern Analysis

**Figure 6:**
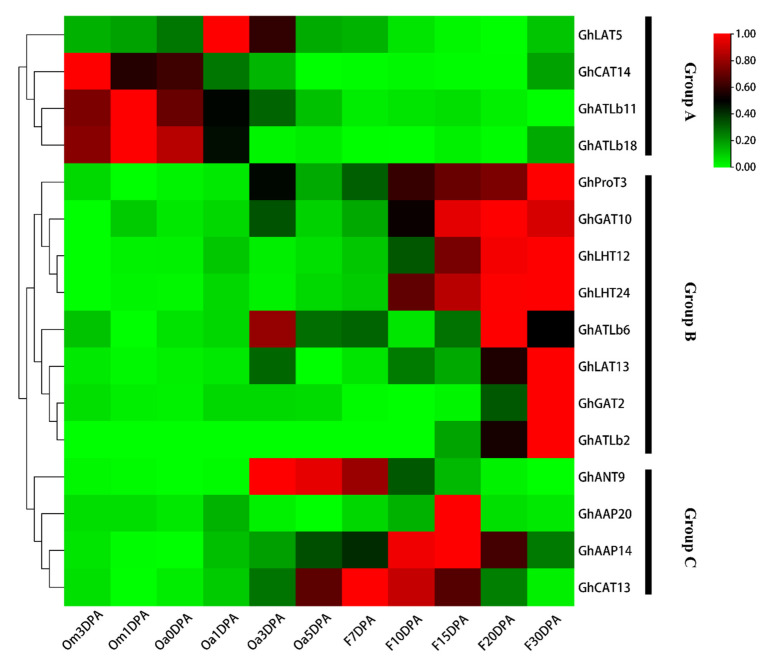
Expression profiles of *GhAATs* during fiber development. The fiber development stages were shown on the bottom; the gene names are shown on the right. This heatmap was clustered into three groups (A–C), which are marked with different colors. Scale bars at the right represented log2 (FPKM + 1), Om3DPA: Ovule at minus 3 day post anthesis, Om1DPA: Ovule at minus 1 day post anthesis, Oa0DPA: Ovule at 0 day post anthesis, Oa1DPA: Ovule at 1 day post anthesis, Oa3DPA: Ovule at 3 day post anthesis, Oa5DPA: Ovule at 5 day post anthesis, F7DPA: Fiber at 7 day post anthesis, F10DPA: Fiber at 10 day post anthesis, F15DPA: Fiber at 15 day post anthesis, F20DPA: Fiber at 20 day post anthesis and F30DPA: Fiber at 30 day post anthesis.

Differential expression quantitative analysis FPKM (Fragments Per Kilobase of transcript per Million fragments mapped) refers to the number of fragments per thousand bases of a gene per million fragments, which is related to the length and expression level of transcripts, and can usually be used as transcript frequency or gene expression level. Among the 190 *GhAAT* family genes, 121 genes were detected with FPKM > 1 in at least one stage of the 11 stages of fiber development. Among the 121 genes, 16 genes were specifically expressed during fiber development, and the other 105 genes were nonspecific. According to their expression patterns, 16 genes are grouped into three groups (a, b and c). Four *GhAAT* genes in group A, GhLAT5, GhCAT14, GhATLb11, GhATLb18, tend to be expressed in the early stage of ovule development (−3 DPA~1 DPA). There were 8 *GhAAT* genes in group B, GhProT3, GhGAT10, GhLHT12, GhLHT24, GhATLb6, GhLAT13, GhGAT2, GhATLb2, which were mainly expressed at fiber elongation and maturation stage (15 DPA~30 DPA). There were four *GhAAT* genes in group C, GhANT9, GhAAP20, GhAAP14, GhCAT13, which tended to be expressed at fiber initiation and elongation (3~15 DPA) (Figure 6). We also randomly selected five genes for qPCR analysis. On the basis of transcriptomic data and qPCR data, we confirm once again that GhProT3, GhLHT12, and GhLHT24 were mainly expressed at fiber elongation and maturation stage (15 DPA~30 DPA). Moreover, GhATLb11 and GhATLb18 were mainly expressed in the early stage of ovule development (−3DPA~1 DPA). The expression trends of these genes during fiber development by qPCR verification were consistent with those predicted by transcriptome data.

### 3.8. Analysis of GhAATs Promoter Regions

To illuminate the regulation network of the 16 specific expressed GhAATs, the 2000 bp upstream regions from the transcript start site (TSS) of the 16 specific expressed and 105 nonspecific expressed GhAATs during fiber development were extracted and analyzed by homer software. The promoters of the 105 nonspecific expressed GhAATs were used as a background, and 23 conserved motifs were identified in most of the target gene promoters and not in the background sequences. Previous studies have shown that bHLH, homeobox, MYB, and WRKY transcription factors are related to cotton fiber development [46]. Among the 23 conserved motifs, motif 7 was predicted to be bHLH transcription factor binding site, motif 6 and motif 9 were predicted to be homeobox transcription factor binding sites, motif 13 was predicted to be WRKY transcription factor binding site, and motif 23 was the MYB transcription factor binding site (Table S22). These results suggested that bHLH, homeobox, MYB and WRKY transcription factors might bind to the promoters of GhAATs, leading to their specific expression during fiber development.

## 4. Discussion

*AAT* family plays an essential role in plant growth and development and resistance to stress [53]. However, it has not been reported that the *AAT* family is involved in cotton fiber development. In this study, the function and expression pattern of the *AAT* genes in *G. hirsutum* L. were analyzed systematically, and the characteristics of *GhAATs* were analyzed at the genome level. In the present study, we identified and characterized members of the *AAT* gene family in cotton through genome-wide analyses and studied their evolutionary model and gene expression patterns during cotton fiber development. This study will help us better understand the *GhAAT* genes and further study their function in the future.

According to previous reports, there are 85 *AAT* genes in rice (*Oryza sativa* L.) [15], 296 in wheat (*Triticum aestivum* L.) [9], 107 in maize (*Zea mays* L.) [16], 189 in soybean (*Glycine max* L.) [14], 63 in *A. thaliana* [15], and 72 in potato (*Solanum tuberosum* L.) [2]. Compared with other reported plants, allotetraploid cotton had more numerous amino acid transporter genes, resulting from its allotetraploid genome and complex evolution. For example, a total of 190 *AAT* genes were identified in cotton, and the number of *GhAAT* genes was much more than that of other plants. Still, the number of *AAT* family genes of *G. hirsutum* L. and *G. barbadense* L. was the same as that of allotetraploid soybean (*Glycine max* L.) (Supplementary Tables S1 and S2) [14], which indicated that the *AAT* family genes of allotetraploid *G. hirsutum* L. and *G. barbadense* L. expanded in the process of evolution like other polyploid species [9]. Moreover, the number of *AAT* genes in two allotetraploid cotton species, *G. hirsutum* L. (Gh) and *G. barbadense* L. (Gb) were about twice as many as *G. arboreum* L. (Ga) and *G. raimondii* L. (Gr) (Supplementary Tables S1–S4). These results indicated that the *AAT* gene family in allotetraploid cotton maybe undergo a large-scale expansion in the process of evolution [9,68,70].

The gene structure and GhAAT protein motifs showed remarkable changes. Still, the members of the same clade showed relative conservancy of gene structure in gene structure and protein motifs, which provided some reference for follow-up functional analysis (Figure 5). This also confirms that the *AAT* gene comes from the same ancestor and may gradually evolve or and their amount in the genome may amplify.

The event of gene segmental duplication is significant in the process of gene family amplification [71,72]. Previous studies in other plants have found that segmental duplication events are the main driving force for the evolution of *AAT* genes [9,73]. In this study, it was found that most of the *AAT* genes in the four cotton species showed segmental duplication. Still, few tandem repeats were found in cotton chromosomes, indicating that segmental duplication plays a critical role in the amplification of the cotton *AAT* genes (Supplementary Tables S7–S14) [74]. Taking *G. hirsutum* L. as an example, the collinear analysis of *G. hirsutum* L. *GhAAT* genes showed 202 pairs of segmental duplication and nine pairs of tandem repeats (Supplementary Tables S7 and S8). Based on these results, it was speculated that in polyploidy, paralogous gene pairs are usually produced from segmental duplication, and segmental duplication is the most critical factor in evolution. There were genome-wide duplication gene pairs between *G. arboreum* and *G. raimondii* (Supplementary Table S19). We speculated that these genes might be present in *G. arboreum* L. and *G. raimondii* L. before the differentiation of genome A and D.

Our results found that some *AAT* genes in the allotetraploid subgenome A or subgenome D were collinear with those in the diploid *G. arboreum* L. (A genome) or *G. raimondii* L. (D genome), respectively. However, some *AAT* genes in subgenome A or subgenome D were collinear with those in diploid *G. raimondii* L. (D genome) or *G. arboreum* L. (A genome)*,* respectively (Figure 4). We speculated that it might be that transposition events occurred between subgenome A and subgenome D after the formation of allotetraploid cotton.

According to the sequence homology and classification of *A. thaliana AAT* family genes [1], the 190 GhAAT proteins in *G. hirsutum* L. can be divided into 13 groups (Figure S1). The AAT proteins from *A. thaliana* and *G. hirsutum* L. were distributed on the same clade confirming that the main features of the *AAT* family had formed before these species differentiated (Figure 1). Similarly, the *AAT* family genes of diploid cotton, *G. arboreum* L., and *G. raimondii* L. were split into 12 groups by phylogenetic analysis (Figure S2). These data suggest that AAT protein grouping was formed before diploid cotton evolved into tetraploid cotton [9]. The classification of the GhAAT family was further confirmed by protein conserved motif analysis. There are significant differences in the number and distribution of conserved motif of *AAT* genes in different groups. In some groups, there are some unique motif, almost conserved motifs, and the conserved motif of AAT proteins in the same clade are highly similar, which indicates that these motifs are related to the phylogeny of *AAT* genes (Figure 5). Although the conserved motifs of GhAAT proteins are similar, there are also many differences in chemical and physical characteristics (Supplementary Table S1). These differences may be due to the amino acid differences in the non-conserved regions of GhAAT proteins, which means that different GhAAT proteins may evolve in different rates.

We analyzed the homologous sequence variation of four cotton species by calculating the replacement rates of non-synonymous (Ka) and synonymous (Ks). The results showed that the Ka/Ks ratio of more than 95% of the homologous gene pairs of *AAT* genes in four cotton species was less than one (Supplementary Table S25), indicating a strong purifying or stabilizing selection in the evolution process of *AAT* genes in genus *Gossypium* and the genes evolution in these plants was slow [75]. We found GhAAP16, GhAAP18, GhANT4, GhANT9, GhATLa2, GhATLb19, GhATLb20, GhATLb4, GhATLb5, GhATLb6, GhATLb9, GhCAT12, GhCAT13, GhCAT9, GhGAT5, GhGAT9, GhLAT13, GhLAT5, GhLHT12, GhLHT4, GhProT2 and GhTTP2 were positive selected. GhLHT12, GhANT9, GhATLb6, GhLAT5, GhLAT13, GhCAT13 tend to express specifically during fiber development (Supplementary Tables S1 and S26). These results suggest that the genes with positive selection may have experienced functional differentiation.

**Figure 7:**
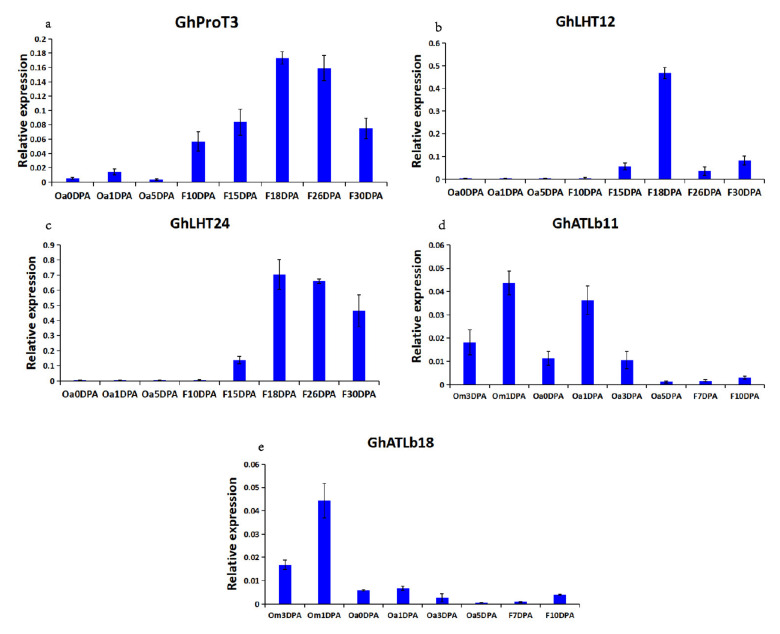
Expression patterns of *AATs* in different fiber development stages via RT qPCR. (**a**–**e**) Represent the expression patterns of *GhProT3, GhLHT12, GhLHT24, GhATLb11, GhATLb18* genes in different fiber development stages. Note: −3 DPA to 30 DPA indicated by −3, −1, 0, 1, 3, 5, 7, 10, 15, 18, 26, 30 DPA. The error bars showed the standard deviation of three biological replicates.

Gene expression pattern analysis can promote the identification of gene function. To study the expression profile of *AAT* genes during fiber development in *G. hirsutum* L., ovules or fibers at different developmental stages were sampled. The transcriptome analysis of the *GhAAT* genes was carried out in *G. hirsutum* L. (Supplementary Table S26). The results showed that among the 190 *GhAAT* family genes, 121 genes were expressed during fiber development, and 16 genes were specifically expressed during fiber development, while the other 105 genes were nonspecific (Figure 6). To verify the reliability of the transcriptome data, we selected 5 of the 16 genes for qPCR verification, and the expression trends were consistent with the transcriptome data (Figure 7). Among the 16 genes specifically expressed during fiber development, four genes tended to be expressed at the early stage of ovule development (−3 DPA~1 DPA), eight genes were mainly expressed at fiber elongation and maturation stage (15 DPA~30 DPA), and four genes tended to be expressed at fiber initiation and fiber elongation stage (3~15 DPA). It is worth noting that there are differences in the process of phylogeny of some *GhAAT* genes, which are closely related to the expression levels during fiber development. For example, among the 16 genes specifically expressed during fiber development, the ATLb group contains the most (four), but they are expressed explicitly at different fiber development stages. *GhATLb11* and *GhATLb18* tend to express specifically at the fiber initiation stage, while *GhATLb6* and *GhATLb2* genes are mainly expressed at the fiber elongation and maturity stage. These results suggest that GhATLb group genes may play an important role in fiber development and may be involved in the whole process from initiation, growth to maturation of cotton fibers.

By analyzing the promoters of *AAT* family genes that are highly expressed during *G. hirsutum* L. fiber development, we identified binding sites for transcription factors that play an important role in fiber development, such as bHLH, Homeobox, MYB, and WRKY transcription factors. These results suggest that the highly expressed *AAT* genes are likely to be regulated by these transcription factors and participate in the biological process of cotton fiber development (Supplementary Table S7).

## 5. Conclusions

To sum up, we identified 190, 190, 101, and 94 full-length *AAT* genes from *G. hirsutum* L., *G. barbadense* L., *G. arboreum* L., and *G. raimondii* L., respectively, and systematically analyzed the *AAT* family genes, including gene evolution, gene structure, protein motif, collinear relationship, cis-acting elements, GO, KEGG, and gene expression patterns. Besides, we also obtained the expression patterns of *AAT* genes during fiber development in *G. hirsutum*. We found 16 genes specifically expressed during fiber development, of which four genes tended to be expressed explicitly at the fiber initiation stage, eight genes tended to be expressed explicitly at the fiber elongation and maturity stage. Four genes tended to be expressed explicitly at the fiber initiation and elongation stage (3~15 DPA). These results laid a foundation for further elucidating the biological function of the *AAT* gene in cotton, especially during cotton fiber development. These results also laid a foundation for further studying the molecular mechanism of the *AAT* gene during fiber development in *G. hirsutum* L.

## Data Availability

The data presented in this study are available in the article and Supplementary Materials.

## Supplementary Materials

The following are available online at https://www.mdpi.com/article/10.3390/plants10112461/s1. Figure S1: Phylogenetic analysis and subfamily classification of the AAT proteins in cotton (GhAATs). Figure S2: Phylogenetic tree of *AAT* proteins in *G. hirsutum* L., *G. barbadense* L., *G. arboreum* L., *G. raimondii* L. Figure S3: Chromosomal distribution of *AATs* in *G. barbadense* L. Figure S4: Chromosomal distribution of *AATs* in *G. arboreum* L. Figure S5: Chromosomal distribution of *AATs* in *G. raimondii* L. Table S1: Identification of *AAT* genes in *G. hirsutum* L. Table S2: Identification of *AAT* genes in *G. barbadense* L. Table S3: Identification of *AAT* genes in *G. arboreum* L. Table S4: Identification of *AAT* genes in *G. raimondii* L. Table S5: Identification of *AAT* genes in *A. thaliana.* Table S6: The *AAT* gene numbers distributed on each chromosome in Gossypiums. Table S7: The segmental duplication gene pairs in *G. hirsutum* L. Table S8: The tandem repeats gene pairs in *G. hirsutum* L. Table S9: The segmental duplication gene pairs in *G. barbadense* L. Table S10: The tandem repeats gene pairs in *G. barbadense* L. Table S11: The segmental duplication gene pairs in *G. arboreum* L. Table S12: The tandem repeats gene pairs in *G. arboreum* L. Table S13: The segmental duplication gene pairs in *G. raimondii* L. Table S14: The tandem repeats gene pairs in *G. raimondii* L. Table S15: The homologous *AAT* gene pairs between *G. arboreum* L. and *G. hirsutum* L. Table S16: The homologous *AAT* gene pairs between *G. raimondii* L. and *G. hirsutum* L. Table S17: The homologous *AAT* gene pairs between *G. arboreum* L. and *G. barbadense* L. Table S18: The homologous *AAT* gene pairs between *G. raimondii* L. and *G. barbadense* L. Table S19: The homologous *AAT* gene pairs between *G. arboreum* L. and *G. raimondii* L. Table S20: The homologous *AAT* gene pairs between *G. hirsutum* L. and *G. barbadense* L. Table S21: The number of genome-wide duplication gene pairs between four *Gossipium* species. Table S22: The conserved motifs in the promoters of 16 specific expressed *GhAATs* in fiber development. Table S23: A list of primers used in RT qPCR experiments. Table S24: Genes matched KEGG pathway items in *G. hirsutum* L. Table S25: The *AAT* genes’ replacement rates of non-synonymous (Ka) and synonymous (Ks) in four cotton species. Table S26: The FPKM value of all genes in *G. hirsutum* L. cultivar Texas Marker-1 (TM-1) during fiber development. Table S27: The result of GO analysis in *G. hirsutum* L.

## Abbreviations

AAT	amino acid transporter
−3 DPA or Om3DPA	Ovule at minus 3 day post anthesis
−1 DPA or Om1DPA	Ovule at minus 1 day post anthesis
0 DPA or Oa0DPA	Ovule at 0 day post anthesis
1 DPA or Oa1DPA	Ovule at 1 day post anthesis
3 DPA or Oa3DPA	Ovule at 3 day post anthesis
5 DPA or Oa5DPA	Ovule at 5 day post anthesis
7 DPA or F7DPA	Fiber at 7 day post anthesis
10 DPA or F10DPA	Fiber at 10 day post anthesis
15 DPA or F15DPA	Fiber at 15 day post anthesis
18 DPA or F18DPA	Fiber at 18 day post anthesis
20 DPA or F20DPA	Fiber at 20 day post anthesis
26 DPA or F26DPA	Fiber at 26 day post anthesis
30 DPA or F30DPA	Fiber at 30 day post anthesis.
*G.*	*Gossypium*
Ka	The replacement rate of non-synonymous
Ks	The replacement rate of synonymous
RT qPCR	Reverse transcription quantitative real-time PCR
FPKM	Fragments Per Kilobase of transcript per Million fragments mapped
GhAt	A genome of *G. hirsutum* L.
GhDt	D genome of *G. hirsutum* L.*G.*
GbAt	A genome of *G. barbadense* L.
GbDt	D genome of *G. barbadense* L.
*A. thaliana*	Arabidopsis thaliana
