# Supplementary material for: Genome-Wide Analysis of AAT Genes and Their Expression Profiling during Fiber Development in Cotton
Source: Plants (Basel). 2021 Nov 15;10(11):2461. doi: 10.3390/plants10112461 (PMC8619630; doi:10.3390/plants10112461)
Supplement: Supplementary file 1 [file plants-10-02461-s001.zip › Additional file1.pdf]

```python
# -*- coding: utf-8 -*-
"""
Created on Thu May  7 11:00:21 2020

@author: liushang
"""

import pandas as pd
import numpy as np
import matplotlib.pyplot as plt
import seaborn as sns
data=pd.read_csv('FPKM.csv',sep='\t',index_col='name')
data=np.log2(data+1)
#trimed all low expressed genes
data_spe_cal=data.loc[data.max(axis=1)>=1]
data_no_spe_cal=data.loc[data.max(axis=1)<1]
#calculate specific scores
spe_matrix=pd.DataFrame()
columns=data_spe_cal.columns.tolist()
for i in columns:
    relate_abundance=np.divide(data_spe_cal[i],data_spe_cal.sum(axis=1))
    spe_matrix[i]=np.multiply(relate_abundance,np.log2(relate_abundance))
spe_matrix=spe_matrix.where(spe_matrix.notnull(),0)
spe_matrix['total']=np.log2(11)
spe_matrix['spe']=spe_matrix['total']+spe_matrix[columns].sum(axis=1)
#select genes according to specific scores
spe_matrix_select=spe_matrix.loc[spe_matrix['spe']>=0.5]
specific=[i for i in spe_matrix_select.index]
non_specific=[]
for i in data_spe_cal.index.tolist():
    if i not in specific:
        non_specific.append(i)
specific_data=data_spe_cal.loc[data_spe_cal.index.isin(specific)]
non_specific_data=data_spe_cal.loc[data_spe_cal.index.isin(non_specific)]
non_specific_data=np.log2(non_specific_data+1)
specific_data=np.log2(specific_data+1)



#plot
plt.figure(figsize=(25,10),dpi=300)
sns.clustermap(non_specific_data,method ='average',metric='euclidean',cmap='Greens',
               linewidths=0.3,alpha=0.6,col_cluster=False)
plt.savefig('non_specific.pdf',bbox_inches='tight')
```

```
plt.clf()
plt.figure(figsize=(25,10),dpi=300)
sns.clustermap(specific_data,method ='average',metric='euclidean',cmap='Greens',
                    linewidths=0.3,alpha=0.6,col_cluster=False)
plt.savefig('specific.pdf',bbox_inches='tight')
plt.clf()
with open('specific_name.txt','w') as file:
    for i in specific:
        file.write(i+'\n')
with open('non_specific_name.txt','w') as file:
    for i in non_specific:
        file.write(i+'\n')
```
