# Supplementary material for: Genome-Wide Analysis of AAT Genes and Their Expression Profiling during Fiber Development in Cotton
Source: Plants (Basel). 2021 Nov 15;10(11):2461. doi: 10.3390/plants10112461 (PMC8619630; doi:10.3390/plants10112461)
Supplement: Supplementary file 1 [file plants-10-02461-s001.zip › Figure S1.pdf]

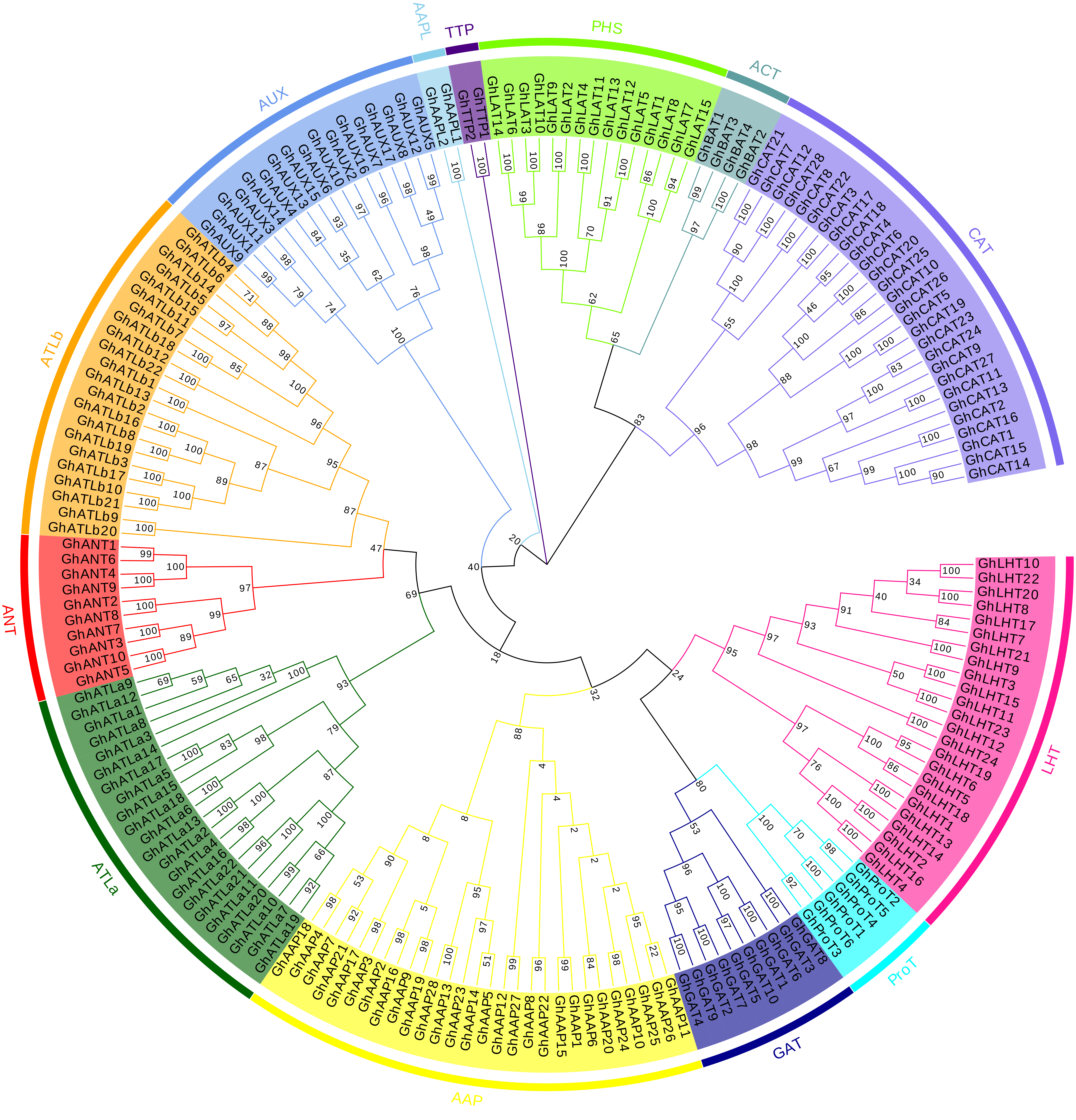
AUX
AAPL
TTP
PHS
ACT
CAT
LHT
ProT
GAT
AAP
ATLa
ANT
ATLb
GhAUX5 GhAUX12 GhAUX8 GhAUX17 GhAUX7 GhAUX16 GhAUX2 GhAUX10 GhAUX6 GhAUX13 GhAUX15 GhAUX4 GhAUX14 GhAUX3 GhAUX11 GhAUX9
GhAAPL2 GhAAPL1
GhTTP1 GhTTP2
GhLAT14 GhLAT6 GhLAT3 GhLAT10 GhLAT9 GhLAT2 GhLAT4 GhLAT11 GhLAT13 GhLAT12 GhLAT5 GhLAT1 GhLAT8 GhLAT7 GhLAT15
GhBAT1 GhBAT3 GhBAT4 GhBAT2
GhCAT21 GhCAT7 GhCAT12 GhCAT28 GhCAT8 GhCAT22 GhCAT3 GhCAT17 GhCAT18 GhCAT4 GhCAT6 GhCAT20 GhCAT25 GhCAT10 GhCAT26 GhCAT5 GhCAT19 GhCAT23 GhCAT24 GhCAT9 GhCAT27 GhCAT11 GhCAT13 GhCAT2 GhCAT16 GhCAT1 GhCAT15 GhCAT14
GhLHT10 GhLHT22 GhLHT20 GhLHT8 GhLHT17 GhLHT7 GhLHT21 GhLHT9 GhLHT3 GhLHT15 GhLHT11 GhLHT23 GhLHT12 GhLHT24 GhLHT19 GhLHT6 GhLHT5 GhLHT18 GhLHT1 GhLHT13 GhLHT14 GhLHT2 GhLHT16 GhLHT4
GhProT2 GhProT5 GhProT4 GhProT1 GhProT6 GhProT3
GhGAT8 GhGAT3 GhGAT6 GhGAT1 GhGAT10 GhGAT5 GhGAT7 GhGAT2 GhGAT9 GhGAT4
GhAAP11 GhAAP26 GhAAP25 GhAAP10 GhAAP24 GhAAP20 GhAAP6 GhAAP1 GhAAP15 GhAAP22 GhAAP8 GhAAP27 GhAAP12 GhAAP5 GhAAP14 GhAAP23 GhAAP13 GhAAP28 GhAAP19 GhAAP9 GhAAP16 GhAAP2 GhAAP3 GhAAP17 GhAAP21 GhAAP7 GhAAP4 GhAAP18
GhATLa19 GhATLa7 GhATLa10 GhATLa20 GhATLa11 GhATLa21 GhATLa22 GhATLa16 GhATLa4 GhATLa2 GhATLa13 GhATLa6 GhATLa18 GhATLa15 GhATLa5 GhATLa17 GhATLa14 GhATLa3 GhATLa8 GhATLa1 GhATLa12 GhATLa9
GhANT5 GhANT10 GhANT3 GhANT7 GhANT8 GhANT2 GhANT9 GhANT4 GhANT6 GhANT1
GhATLb20 GhATLb9 GhATLb21 GhATLb10 GhATLb17 GhATLb3 GhATLb19 GhATLb8 GhATLb16 GhATLb2 GhATLb13 GhATLb1 GhATLb22 GhATLb12 GhATLb18 GhATLb7 GhATLb11 GhATLb15 GhATLb5 GhATLb14 GhATLb6 GhATLb4

Figure S1. Phylogenetic analysis and subfamily classification of the AAT proteins in cotton (GhAATs). The phylogenetic tree was constructed with MEGAX software using the maximum likelihood (ML) method with 1000 bootstrap replicates. All 190 GhAATs were divided into thirteen groups which were highlighted by different colors (AAP, LHT, GAT, ProT, AUX, ATLa, ANT, ATLb, AAPL, TTP, PHS, ACT, and CAT).
