# Supplementary material for: Genome-Wide Analysis of AAT Genes and Their Expression Profiling during Fiber Development in Cotton
Source: Plants (Basel). 2021 Nov 15;10(11):2461. doi: 10.3390/plants10112461 (PMC8619630; doi:10.3390/plants10112461)
Supplement: Supplementary file 1 [file plants-10-02461-s001.zip › Figure S2.pdf]

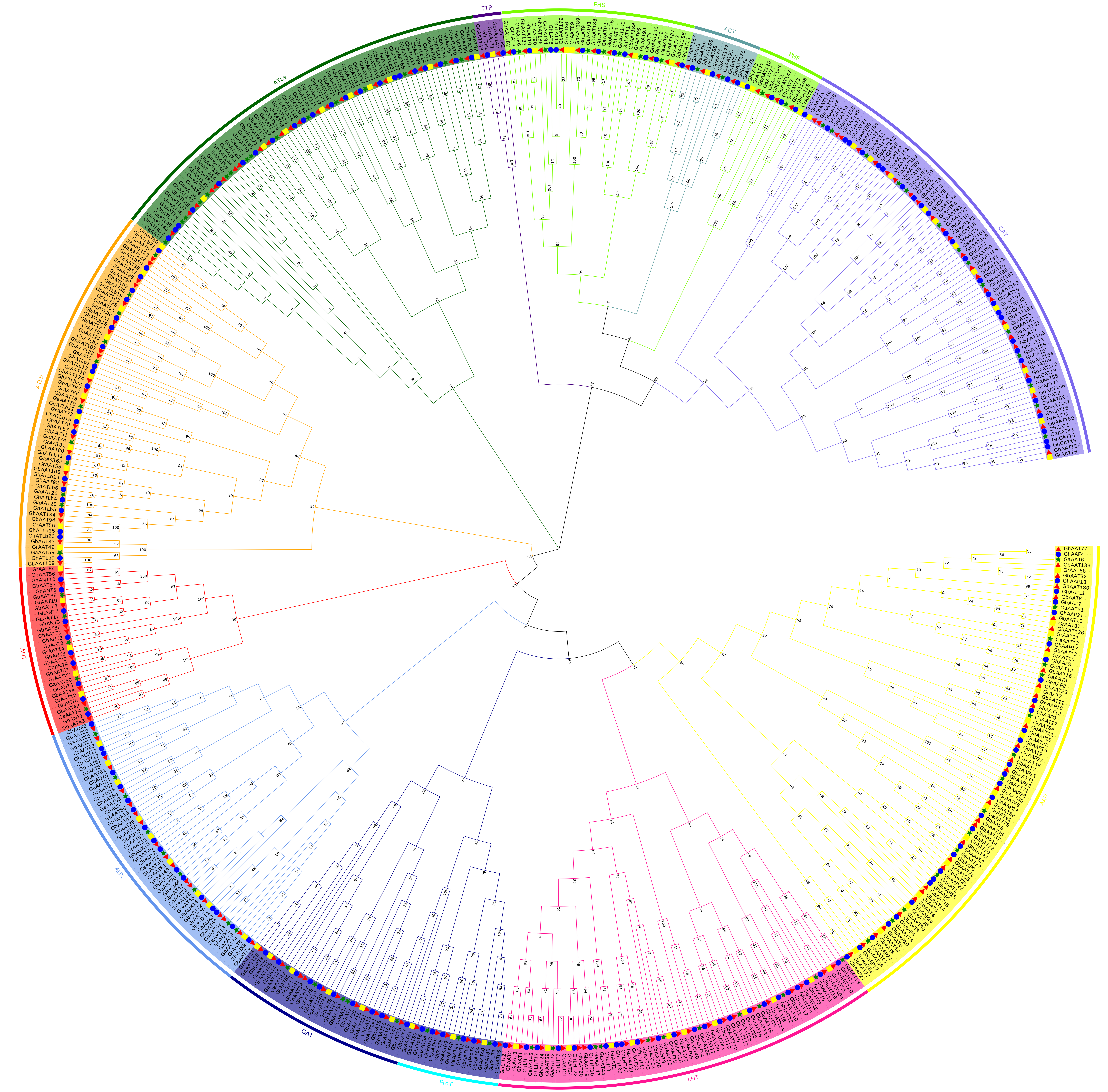
TTP
PHS
ACT
PHS
CAT
AAP
LHT
ProT
GAT
AUX
ANT
ATLb
ATLa

Figure S2. Phylogenetic tree of *AAT* proteins in *G. hirsutum* L., G. barbadense L., G. arboreum L., *G. raimondii* L.. 190 GhAATs from *G. hirsutum* L. marked by blue circles, 190 GbAATs from G. barbadense L. marked by red triangles, 101 GaAATs from G. arboreum L. marked by green star and 94 GrAATs from *G. raimondii* L. marked by yellow rect. The phylogenetic tree was generated using MEGAX via the maximum likelihood (ML) method with 1000 bootstrap replicates. All AAT proteins were divided into 12 subgroups which were highlighted by different colors (AAP, LHT, GAT, ProT, AUX, ATLa, ANT, ATLb, TTP, PHS, ACT, and CAT).
