# Supplementary material for: Genome-Wide Analysis of AAT Genes and Their Expression Profiling during Fiber Development in Cotton
Source: Plants (Basel). 2021 Nov 15;10(11):2461. doi: 10.3390/plants10112461 (PMC8619630; doi:10.3390/plants10112461)
Supplement: Supplementary file 1 [file plants-10-02461-s001.zip › Figure S3.pdf]

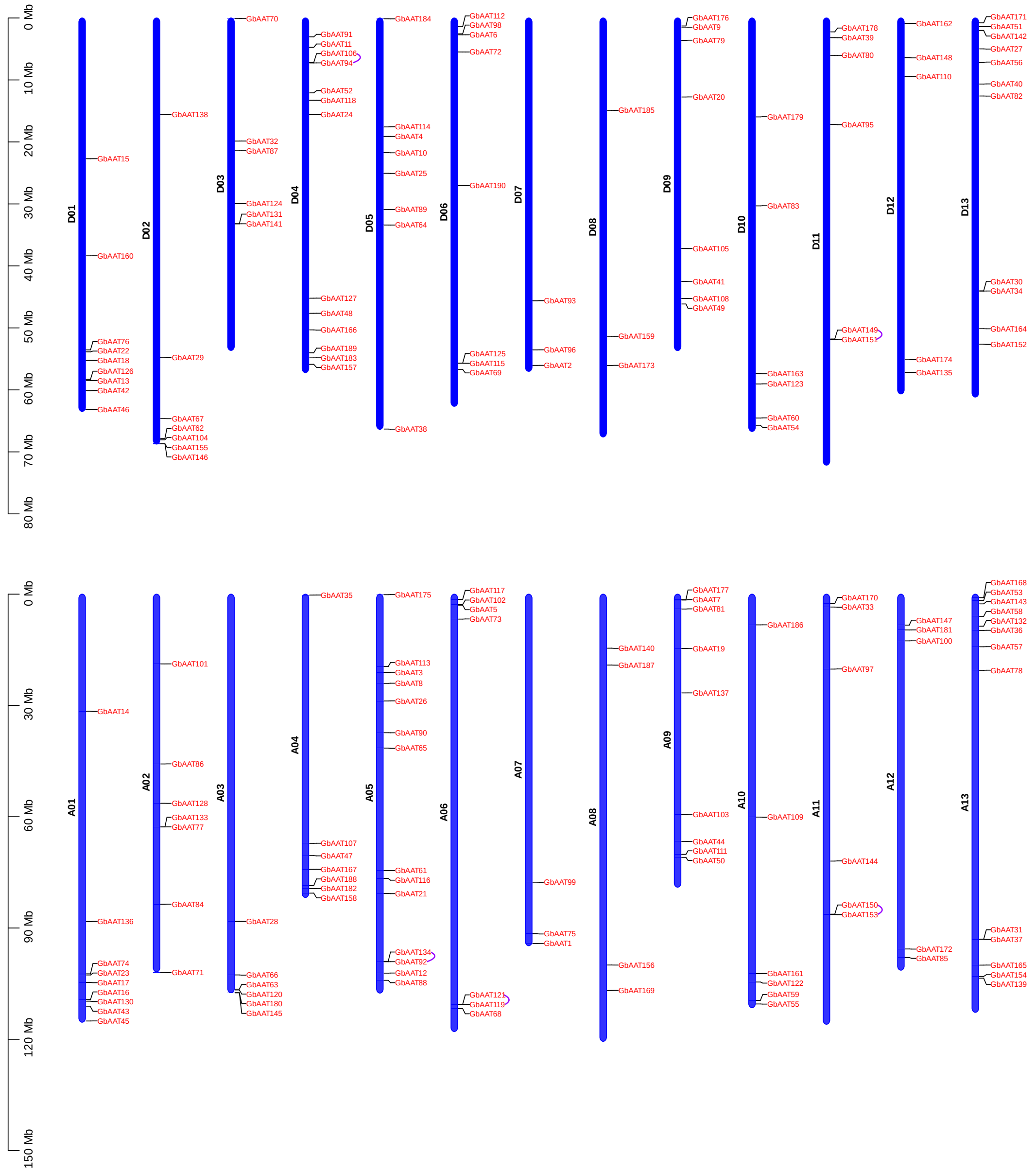

Figure S3. Chromosomal distribution of *AATs* in *G. barbadense* L. The chromosome IDs were indicated beside each vertical bar. The *AATs* were displayed on different chromosomes. Blue, bars represented the physical maps of chromosomes in *G. barbadense* L.
