# Supplementary figures and images for: Genome-Wide Analysis of AAT Genes and Their Expression Profiling during Fiber Development in Cotton
Source: Plants (Basel). 2021 Nov 15;10(11):2461. doi: 10.3390/plants10112461 (PMC8619630; doi:10.3390/plants10112461)

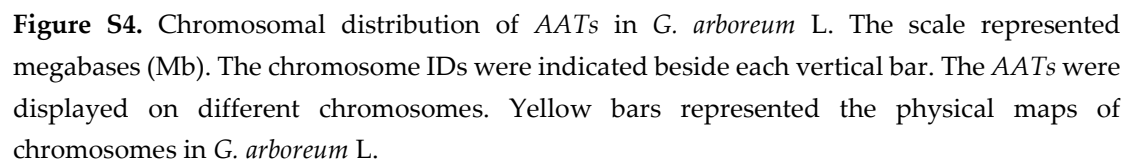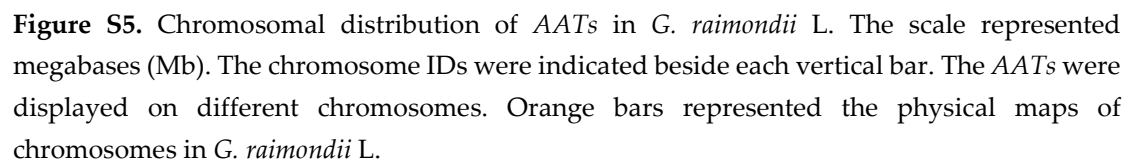

Supplement: Supplementary file 1 [file plants-10-02461-s001.zip › Figure S4 and S5.pdf]
